# All-Suture Anchor vs. Knotless Suture Anchor for the Treatment of Anterior Shoulder Instability—A Prospective Cohort Study

**DOI:** 10.3390/jcm13051381

**Published:** 2024-02-28

**Authors:** Marvin Minkus, Annette Aigner, Julia Wolke, Markus Scheibel

**Affiliations:** 1 Department for Shoulder and Elbow Surgery, Center for Musculoskeletal Surgery, Charité Universitaetsmedizin Berlin, 10117 Berlin, Germany; marvin.minkus@charite.de (M.M.);; 2 Institute of Biometry and Clinical Epidemiology, Charité Universitaetsmedizin Berlin, 10117 Berlin, Germany; 3 Department of Shoulder and Elbow Surgery, Schulthess Clinic Zurich, 8008 Zurich, Switzerland

**Keywords:** shoulder instability, bankart repair, all-suture anchor

## Abstract

All-suture or soft-anchors (SA) represent a new generation of suture anchor technology with a completely suture-based system. This study’s objective was to assess Juggerknot^®^ SA, for arthroscopic Bankart repair in recurrent shoulder instability (RSI), and to compare it to a commonly performed knotless anchor (KA) technique (Pushlock^®^). In a prospective cohort study, 30 consecutive patients scheduled for reconstruction of the capsulolabral complex without substantial glenoid bone loss were included and operated on using the SA technique. A historical control group was operated on using the KA technique for the same indication. Clinical examinations were performed preoperatively and 12 and 24 months postoperatively. RSI and WOSI at 24 months were the co-primary endpoints, evaluated with logistic and linear regression. A total of 5 out of 30 (16.7%) patients suffered from RSI in the SA group, one out of 31 (3.2%) in the KA group (adjusted odds ratio = 10.12, 95% CI: 0.89–115.35), and 13.3% in the SA group and 3.2% in the KAgroup had a revision. The median WOSI in the SA group was lower than in the KA group (81% vs. 95%) (adjusted regression coefficient = 10.12, 95% CI: 0.89–115.35). Arthroscopic capsulolabral repair for RSI using either the SA or KA technique led to satisfying clinical outcomes. However, there is a tendency for higher RSI and lower WOSI following the SA technique.

## 1. Introduction

Anterior shoulder instability is common in the general population, especially in young and physically active persons. The arthroscopic shoulder stabilization with refixation of the labrum is a standard procedure for patients with symptomatic anteroinferior shoulder instability without glenoid bone loss. Numerous fixation techniques and devices are available for conducting the so-called Bankart procedure. In general, two different types of surgical procedures can be distinguished: the suture-first and the anchor-first technique. The respective anchors have different characteristics regarding refixation of the capsulolabral complex. The suture-first technique, where first sutures are shuttled through the labrum, requires anchors connected to the sutures secondarily and can then be punched into the glenoid in terms of a knotless fixation. The anchor-first technique requires suture anchors placed at the anteroinferior labrum first and then the sutures are shuttled through the labrum. Furthermore, biodegradable and nonabsorbable anchors are distinguishable. Metallic anchors have largely been replaced, due to concerns of articular cartilage damage, migration, and loosening [1]. The development of osteolysis around anchors is always a matter of concern and has been reported for both biocomposite and all-suture anchors [2,3]. All-suture or soft-anchors represent a new generation of suture anchor technology with a completely suture-based system, which allows for small pilot holes and potentially reduces occurrence of osteolysis.

Only a few studies have compared clinical outcomes of two different anchor techniques in shoulder stabilization surgeries. Lee et al. published a retrospective study with comparison of clinical outcomes after arthroscopic Bankart repair using all-suture anchors and biodegradable suture anchors [4]. Two recurrent dislocations were observed in both groups. The authors found a comparable clinical outcome and postoperative stability at 2 years after surgery. In a prospective randomized controlled trial by Tan et al., no significant differences in outcomes of arthroscopic Bankart repair were found, for both absorbable or nonabsorbable suture anchors. Only a few studies have compared knotless and knot-tying anchors for arthroscopic capsulolabral repair in patients with shoulder instability. In a retrospective matched-pair analysis, Wu et al. found similar rates of recurrent dislocations and revision surgeries, but lower rates of recurrent subluxation in knotless anchors compared to knot-tying anchors [5].

However, there is a need for clinical evidence to substantiate claims on safety and effectiveness with this product compared to commonly used knotless anchor techniques. While several studies have investigated the biomechanical and biodegradable properties of different anchors, only a few studies exist comparing the clinical outcomes in patients who have undergone arthroscopic shoulder stabilization with labral repair. The objective of this study was to assess the safety and efficacy of all-suture anchors (Juggerknot^®^ Soft Anchors, Fa. Zimmer Biomet, Warsaw, IN, USA) for arthroscopic Bankart repair in recurrent shoulder instability and compare it to a commonly performed knotless anchor technique (Pushlock^®^, Fa. Arthrex, Naples, FL, USA).

## 2. Material and Methods

In a prospective observational cohort study, patients with recurrent anteroinferior shoulder instability were screened for eligibility and recruited at our department for shoulder surgery. With the emergence of all-suture anchors and good experiences with other indications (rotator cuff surgery, distal biceps tendon refixation, etc.), these anchors were also applied for capsulolabral repair (Bankart surgery). Patients who were scheduled for surgery with repair of the capsulolabral complex (Bankart repair) were screened according to the inclusion and exclusion criteria mentioned below. If the criteria were met and patients gave their written consent, shoulder stabilization surgery was performed in an anchor-first technique. Patients were examined preoperatively and followed-up 12 and 24 months after intervention. We included patients aged 18 to 40 years, both male and female, with recurrent anteroinferior glenohumeral instability confirmed by magnetic resonance imaging (MRI) and clinical examination. Patients were included if conservative treatment failed, given patient’s agreement for study participation and an indication for arthroscopic shoulder stabilization was evident. Patients were excluded given first time dislocations, previous shoulder surgery, significant bony glenoidal defect, cuff tears, neuromuscular diseases, drug or alcohol abuse, workers compensation cases, irreversible muscle damage, or in case the patient had inflammatory arthritis (rheumatoid arthritis), was immuno-suppressed or had an autoimmune disorder, or an active infection, local or systemic.

Baseline examination included general and shoulder-instability-specific anamnesis (mechanism/trauma leading to shoulder dislocation, time since first dislocation, and previous treatment). Clinical examination included assessment of range of motion (ROM), shoulder instability (apprehension test, joint laxity) with determination of the type of shoulder instability according to Gerber [6] (B2 or B3) and shoulder outcome measurement with subjective and objective clinical shoulder scores in terms of the Subjective Shoulder Value (SSV) [7], Constant–Murley Score (CS) [8], Rowe Score (RS) [9], Walch–Duplay Score (WDS) [10], and Western Ontario Shoulder Instability Index (WOSI) [11,12]. Preoperative imaging included a computed tomography (CT) scan with 3D reconstruction and measuring of the glenoid defect (Pico method) [13,14]. Postoperative follow-up examinations after 12 and 24 months included the aforementioned clinical assessment, and the evaluation of any adverse event including recurrent shoulder instability. Recurrent instability included any traumatic or atraumatic dislocation or subluxation event reported by the patient. Clinical examinations pre- and postoperatively as well as radiological evaluation were performed by two orthopedic doctors (M.M. and J.W.), who were not the surgeon.

The data collected from this prospective cohort using the all-suture anchor Juggerknot^®^ Soft Anchors (Zimmer Biomet, Warsaw, IN, USA) technique were then compared to a prior historic cohort of patients with shoulder instability and comparable baseline characteristics and clinical outcome assessment, which were operated upon using an anchor-first technique using Pushlock^®^ anchors (Arthrex, Naples, FL, USA). This technique was applied in our department before the emergence of all-suture anchors. The two surgical techniques are described below. All surgeries in both groups were performed by the same surgeon (M.S.), who has more than 15 years of experience in shoulder surgery.

The study was approved by the local ethics committee (EA2/076/13).

### 2.1. Surgical Technique

A standard arthroscopic Bankart repair with refixation of the anteroinferior labrum and capsule was performed in both groups. Patients were placed in the lateral decubitus position with the affected arm in a traction device. A diagnostic arthroscopy was performed via a posterior portal. Then an anteroinferior and anterosuperior portal were created and the arthroscope was switched to the anterosuperior portal. Two twist-in cannulas (Arthrex, Naples, FL, USA) were inserted in the anteroinferior and posterior portal for easier shuttling of the sutures. At first the capsule-labrum complex was mobilized and the glenoid rim cleared of soft tissue. For refixation of the labrum, two different kinds of anchors were used. In the SA group, the anchor-first technique was used using all-suture anchors (Juggerknot by Zimmer Biomet, Warsaw, IN, USA). In the KA group, the suture-first technique was applied using knotless suture anchors (Pushlock by Arthrex, Naples, FL, USA). An additional Remplissage was performed in cases of off-track Hill Sachs lesions in both groups.

#### 2.1.1. SA Group: Anchor-First Technique Using All-Sutures Anchors—Juggerknot (Zimmer Biomet, Warsaw, IN, USA)

**Figure 1:**
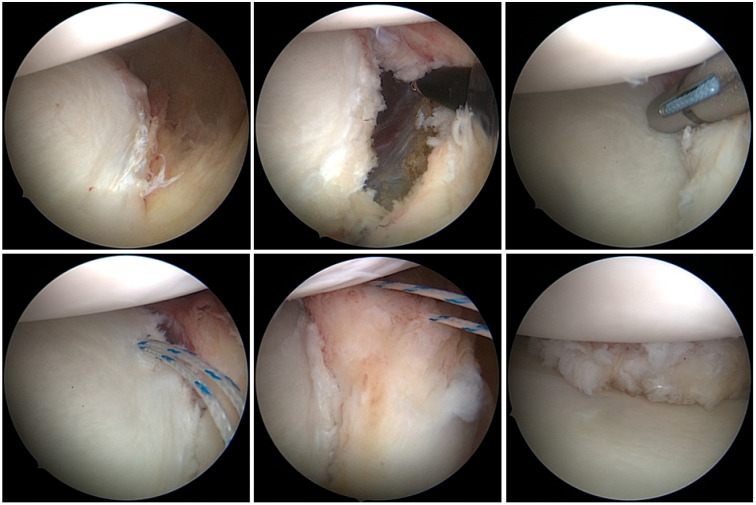
Surgical technique of arthroscopic Bankart repair using all-suture anchors.

Juggerknot soft anchors are available in different sizes and sutures, single- or double loaded. Most commonly used for labral repair are the 1.5 mm and 1.4 mm anchors with MaxBraid™ Sutures (Zimmer Biomet, Warsaw, IN, USA). In this study cohort, the single-loaded 1.4 mm anchors were used. A polyester sleeve and the ZipLoop™ Technology (Zimmer Biomet, Warsaw, IN, USA) allow fixation of soft tissue to bone without any rigid material. Compared to the technique mentioned above, where first sutures are shuttled through the labrum (suture-first), in this surgical technique the anchors are placed first. The Juggerknot guide was passed via the anteroinferior cannula to the 5 to 6 o’clock position of the glenoid. Appropriate depth of the drilling was ensured as the collar of the drill contacted the back of the drill guide. Then the drill was removed, while the guide was held in position. The Juggerknot soft anchor was inserted through the guide into the drill hole. Once the anchor was fully seated into the glenoid bone, the anchor inserter was removed. The sutures were released from the handle and, by pulling on both suture ends, the sleeve forms a clew underneath the cortical bone. Sliding of the suture was still possible, allowing later knot tying. Once the anchor was placed, the sutures were shuttled through the labrum creating a mattress stitch configuration anterior to the labrum (Figure 1). Using a sliding knot and additional reversed-post half-hitched, the capsulolabral tissue was reattached to the glenoid rim. Two additional anchors were placed superior to the first.

#### 2.1.2. KA Group: Suture-First Technique Using Knotless Suture Anchors—Pushlock (Arthrex, Naples, FL, USA)

**Figure 2:**
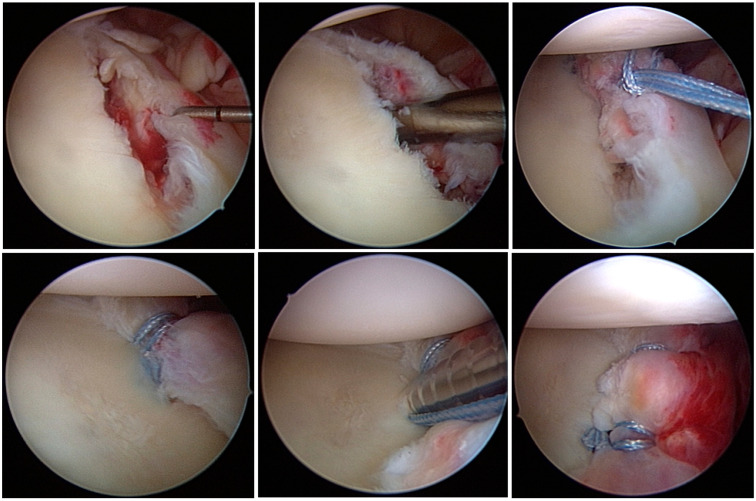
Surgical technique of arthroscopic Bankart repair using knotless suture anchors.

Pushlock^®^ anchors are available in two different materials—Polyether ether ketone (PEEK) and BioComposite. In this patient cohort, only PEEK anchors were used with a diameter of 2.9 mm. At first the capsule-labrum complex at the 5 to 6 o´clock position was penetrated using a SutureLasso™ (Arthrex, Naples, FL, USA). Then a FiberWire™ (Arthrex, Naples, FL, USA) was shuttled through the labrum. Thereby a loop is created, also called a cinch stitch, with the help of the nitinol wire of the SutureLasso (Figure 2). Another Fiberwire was shuttled through the labrum in the same technique just above the first. A drill guide was used for drilling on the desired position at the anteroinferior glenoid. Then the suture ends were passed through the eyelet of the Pushlock anchor, and the anchor was inserted via the anteroinferior portal. The spear was placed onto the glenoid rim. The sutures were tightened and the anchor was advanced into the glenoid. Superior to the first one, two additional anchors were inserted for refixation of the anteroinferior labrum.

#### 2.1.3. Rehabilitation

Rehabilitation protocol was equivalent in both groups. The shoulder was immobilized in a sling in internal rotation for 6 weeks with only limited passive range of motion up to 60° flexion/abduction and 0° external rotation during the first 3 weeks, and 90° flexion/abduction during the following 3 weeks. Active range of motion exercises were initiated 7 weeks postoperatively, followed by muscle strengthening.

### 2.2. Endpoints and Statistical Analysis

The co-primary endpoints for effectiveness were the recurrence of a luxation (yes/no) within the two-year follow-up, and the Western Ontario Shoulder Instability Index (WOSI) score in percent, measuring functional outcomes two years after baseline. Secondary endpoints were the Subjective Shoulder Value (SSV), Constant–Murley Score (CS), Rowe Score (RS), and the Walch–Duplay Score (WD). Safety was measured by assessing all adverse events experienced by patients, which were related or probably related to the study device.

The intention-to-treat (ITT) population included subjects who are correctly included and will be independent from further protocol deviations and violations. This will be the primary cohort. The per-protocol (PP) cohort consists of subjects in whom the study eligibility criteria were strictly respected, who strictly adhered to the procedures defined in the protocol, for whom the primary endpoint was determined, i.e., who completed the study.

To describe baseline characteristics, just as all endpoints were measured at each time point, we report relative and absolute frequencies for categorical variables, and median and interquartile range (IQR) for continuous variables. The co-primary endpoints at 24 months were evaluated using logistic regression for recurrence of a dislocation and linear regression for WOSI (%), adjusting for age at surgery, glenoid defect in percent, whether at least 10 dislocations happened prior to study participation, and whether the affected side was the dominant side. The linear regression model was modelled as the difference between the measurement at t24 and baseline as the dependent variable, adjusting for baseline. Correcting for multiple testing, the effects were considered significant based on a significance level of 2.5%. The secondary endpoints and the WOSI (%) score were evaluated using mixed linear regression, where both follow-up time points (t12 and t24) were taken into account. The dependent variable was defined as the change from t0 to t12 or t24, respectively, additional to the treatment group; independent variables were baseline measurement, age at surgery, glenoid defect in percent, whether at least 10 dislocations happened prior to study participation, and whether the affected side was the dominant side.

Analyses for secondary endpoints were not corrected for multiple testing and *p*-values therefore have to be interpreted as exploratory. Missing values were primarily handled using multiple imputation. As a sensitivity analysis, and corresponding to the per protocol set, we also performed complete-case analyses for all models. Based on these model, odds ratio (OR) and regression coefficients were estimated, along with 95% confidence intervals (CI).

All statistical analyses were performed with the software R version 4.2.3 (The R Foundation, Vienna, Austria), and additional R packages [15,16,17,18].

## 3. Results

### 3.1. Study Population Characteristics

In the SA group, 30 patients (n = 9 female, n = 21 male) with a median age of 29 meeting the inclusion criteria were prospectively enrolled and operated on using the aforementioned technique using the anchor-first technique with all-suture anchors (Juggerknot^®^, Zimmer Biomet, Warsaw, IN, USA). As the historic cohort (KA group), i.e., those operated upon using a suture-first technique using Pushlock^®^ anchors (Arthrex, Naples, FL, USA), 31 patients (n = 6 female, n = 25 male) with a median age of 24 were available (Table 1).

### 3.2. Primary Endpoint Analyses and Adverse events

Overall, six (9.8%) patients suffered from a recurrent dislocation, five (16.7%) in the SA group and one in the KA group (3.2%). Therefore, the revision rate in the SA group was 13.3% and 3.2% in the KA group. The cause for recurrent dislocations was traumatic in all but one case. All traumatic recurrent dislocations occurred within the first year after shoulder stabilization surgery (range 9–12 months) during sportive activities. In the SA group, one patient reported a subluxation during cross-country skiing, which only happened once. Apart from this patient, the other patients (n = 5, 8.1%) required revision surgery (including implant removal) due to persisting shoulder instability and recurrent dislocations. In four cases, a revision soft tissue stabilization was conducted and in one case, a bony augmentation using an iliac crest bone graft was used (prior surgery with all-suture anchor, SA group). No further complications or adverse events were recorded (Table S1, Supplementary Material). Adjusting for age at surgery, glenoid defect in percent, whether at least 10 dislocations happened prior to study participation, and whether the affected side was the dominant side in a logistic regression, the odds for recurrent dislocation were about 10-times higher in the SA group, compared to the KA group (OR = 10.12, 95% CI: 0.89–115.35). Based on the significance level of 2.5%, the difference between the two treatment groups was not significant (Table 2).

The median WOSI score in the SA group was lower than in KA group (81% vs. 95%) at the 24 months follow-up examination. Adjusting for relevant confounders in a linear regression, patients in the KA group had a 12%-points higher WOSI score (regression coefficient = 11.83, 95% CI: 3.59–20.07). Based on the significance level of 2.5%, the difference between the two treatment groups was significant within both the ITT and the PP population (Table 2).

### 3.3. Secondary Endpoint Analyses

**Figure 3:**
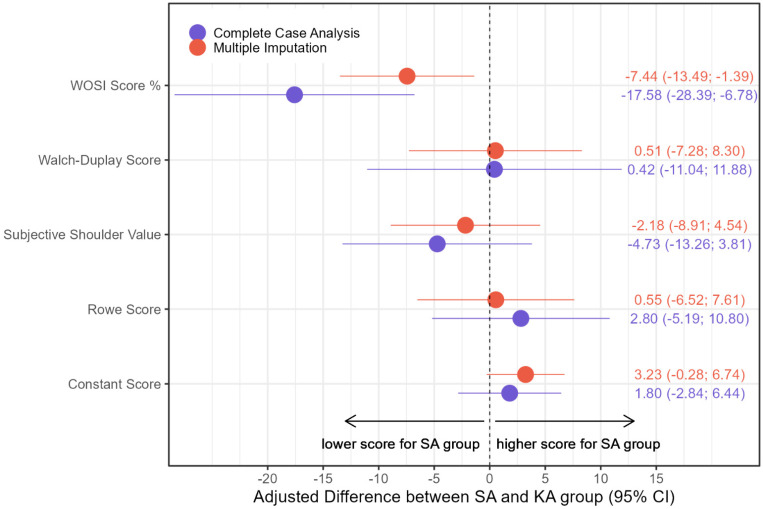
Regression coefficients showing the effect of Juggerknot vs. Pushlock regarding co-primary endpoint WOSI [%] and all secondary endpoints, based on mixed effects linear regression, ITT based on multiple imputation, PP based on complete-case analysis.

Based on mixed effects linear regression models, the WOSI score and the Constant Score differed relevantly between the two treatment groups over all follow-up time points. Based on these models, patients treated with SA are estimated to have a 7.4%-points lower WOSI score (−7.44, 95% CI: −13.49, −1.39), and a 3.2 higher Constant Score (3.23, 95% CI: −0.28, 6.74), based on multiple imputation, in the ITT population. The differences between the groups are irrelevant for the Walch–Duplay Score and not clear for the SSV and Rowe score, where wide confidence intervals indicate great uncertainty in the effect estimation (Figure 3).

## 4. Discussion

The objective of this study was to assess safety and efficacy of all-suture anchors (Juggerknot^®^ Soft Anchors, Fa. Zimmer Biomet) for arthroscopic Bankart repair in recurrent shoulder instability and compare it to a commonly performed knotless anchor technique (Pushlock^®^, Fa. Arthrex). The baseline characteristics of the two cohorts were comparable, apart from a higher WOSI in the KA group. Both surgical techniques and anchors led to satisfying clinical outcomes. However, more recurrent instability events following the all-suture anchor technique were found in this study.

We suggest that the punch mechanism of the knotless anchor technique might enable a higher reduction of the labrum resulting in a bigger glenoidal convavity, which might be favorable for the glenohumeral stability. Furthermore, the learning curve of the all-suture anchor technique might also be an explanation for the higher recurrence and revision rate in this group.

Only few studies compared clinical outcomes of two different anchor techniques in shoulder stabilization surgeries. Lee et al. published a retrospective study with comparison of clinical outcomes and CT analysis of tunnel diameter after arthroscopic Bankart repair using all-suture anchors (group A) and biodegradable suture anchors (group B) [4]. A total of 67 patients with a mean age of 27 years were enrolled and retrospectively analyzed. In group A (n = 33), 1.3 mm single-loaded or 1.8 mm double-loaded all-suture anchors were used and in group B (n = 34) a 3.0 mm biodegradable anchor was used. Baseline characteristics of the patients showed no significant differences. Two recurrent dislocations were observed in both groups. The authors found comparable clinical outcomes and postoperative stability at 2 years after surgery. The tunnel diameter increment was significantly greater in the all-suture anchor patients, but did not influence the clinical outcomes in this study [4]. In a prospective randomized controlled trial by Tan et al., no significant differences in outcomes of arthroscopic Bankart repair were found, for both absorbable or nonabsorbable suture anchors [19]. Only a few studies have compared knotless and knot-tying anchors for arthroscopic capsulolabral repair in patients with shoulder instability. In a retrospective matched-pair analysis, Wu et al. found similar rates of recurrent dislocations and revision surgeries, but lower rates of recurrent subluxation in knotless anchors compared to knot-tying anchors [5].

Not only may the type of anchor used for shoulder stabilization have an influence, but the suture configuration also has an influence on the resulting stability. In a cadaveric and biomechanical study, Miskovsky et al. found that horizontal mattress suture configurations create a larger area of repair, decreasing the risk of repair failure at the labrum compared to simple suture configuration [20]. Similar findings were made by Hagstrom et al. [21]. By different mechanisms of the anchors used in our study, also different types of suture configuration have been applied. For the suture anchors, a lasso-loop stitch was used and a mattress stitch for the all-suture anchors.

The development of osteolysis around anchors is always a matter of concern and has been reported for both biocomposite and all-suture anchors. Stewart et al. compared the bone response of a traditional biocomposite push-fit anchor to an all-suture anchor in MRI scans at 3 weeks and 6 months after labral reconstruction surgery of the shoulder. They found that the all-suture anchors caused significantly less osteolysis in glenoid bone compared to biocomposite anchors [2]. Tompane et al. evaluated follow-up CT scans of 30 patients who underwent arthroscopic shoulder stabilization using all-suture anchors and found low rates of cyst formation after 1, 6, and 12 months, but a significantly increased tunnel volume over time, which was not associated with the initial tunnel location [3]. In our study, no routine follow-up CT scans or MRI scans were performed to evaluate osteolysis.

There are some limitations of our study. We compared two consecutive patient cohorts and no randomization was performed. One group consisted of historic data, which were collected in a routine manner. Furthermore, the learning curve, especially for the all-suture anchor technique, has to be considered, although all surgeries were performed by an experienced shoulder surgeon. The patient cohorts are small and no long-term data were evaluated. The strength of our study is that due to strict inclusion and exclusion criteria and comparable baseline characteristics we were able to compare the two anchor techniques in a defined patient population suffering from recurrent shoulder instability and need for capsulolabral repair. We showed that both techniques can be used for capsulolabral repair and lead to satisfying clinical outcomes, with a tendency for higher recurrent instability for the all-suture anchor technique. We have not been able to clarify the exact mechanism of failure or if these data are confirmed in a longer follow-up period or bigger patient population.

## 5. Conclusions

The arthroscopic capsulolabral repair for recurrent shoulder instability using an all-suture anchor technique and using a knotless anchor technique both lead to satisfying clinical outcomes. However, there is a tendency for higher recurrent instability events following the all-suture anchor technique. The mechanism of failure of all-suture anchors remains unclear and further research on this topic is necessary.

## Figures and Tables

**Table 1:** Baseline characteristics.

	SA Group (n = 30)	KA Group (n = 31)	Total (n = 61)
**Sex**			
Female	9 (30.0%)	6 (19.4%)	15 (24.6%)
Male	21 (70.0%)	25 (80.6%)	46 (75.4%)
**Age at time of surgery**			
Median (IQR)	29 (21, 33)	24 (20, 29)	27 (21, 32)
**Glenoid defect size [%]**			
Median (IQR)	3.4 (3.0, 5.0)	4.0 (2.2, 5.4)	3.8 (2.3, 5.4)
Missing	11 (37%)	1 (3%)	12 (30%)
**>10 dislocations**			
No	20 (71.4%)	25 (80.6%)	45 (76.3%)
Yes	8 (28.6%)	6 (19.4%)	14 (23.7%)
**Dominant side affected**			
Yes	15 (50%)	18 (58.1%)	33 (54.1%)
No	15 (50%)	13 (41.9%)	28 (45.9%)
**Dominant side**			
Left	2 (6.7%)	2 (6.5%)	4 (6.6%)
Right	28 (93.3%)	29 (93.5%)	57 (93.4%)
**Type of instability**			
B2	27 (90%)	15 (48.4%)	42 (68.9%)
B3	3 (10%)	16 (51.6%)	19 (31.1%)
**Additional Remplissage**			
None	30 (100%)	29 (93.5%)	59 (96.7%)
Yes	0 (0%)	2 (6.5%)	2 (3.3%)
**Western Ontario Shoulder Instability Index (WOSI) [%] t0**			
Median (IQR)	40.5 (33.8, 53.2)	51.6 (40.0, 69.5)	44.5 (35.0, 58.0)
**Subjective Shoulder Value (SSV) t0**			
Median (IQR)	57.5 (50.0, 68.8)	50.0 (30.0, 65.0)	50.0 (40.0, 65.0)
**Constant–Murley Score (CS) t0**			
Median (IQR)	73.5 (71.0, 80.8)	77.0 (73.0, 82.5)	76.0 (71.0, 82.0)
**Rowe Score (RS) t0**			
Median (IQR)	15.0 (5.0, 25.0)	15.0 (15.0, 25.0)	15.0 (10.0, 25.0)
**Walch–Duplay Score (WDS) t0**			
Median (IQR)	25.0 (21.3, 33.8)	30.0 (25.0, 40.0)	25.00 (25.0, 40.0)

**Table 2:** Primary outcome parameters by surgery and primary analyses.

	Juggerknot (n = 30)	Pushlock (n = 31)	Total (n = 61)	Effect Estimate (95% Confidence Interval) and *p*-Value	
				Intention to Treat Analysis *	Per Protocol **
**Recurrent dislocation**				Odds ratio = 10.12 (0.89; 115.35) *p*-value = 0.062	Odds ratio = 12.26 (1.46; 275.56) *p*-value = 0.041
No	25 (83.3%)	30 (96.8%)	55 (90.2%)		
Yes	5 (16.7%)	1 (3.2%)	6 (9.8%)		
**WOSI [%] t12**					
Median (IQR)	81.9 (73.1, 86.4)	86 (75.5, 93)	84.6 (74.2, 90)		
Missing	3 (10%)	4 (12.9%)	7 (11.48%)		
**WOSI [%] t24**				Regression coefficient = −11.75 (−19.56; −3.94) *p*-value = 0.004	Regression coefficient = −17.58 (−28.39; −6.78) *p*-value = 0.002
Median (IQR)	80.7 (73, 85.6)	95 (86.3, 97)	86.4 (76.9, 95.3)		

Significance level: 0.025. All analyses adjusted for age at surgery, glenoid defect in percent, whether at least 10 luxations happened prior to study participation, and whether the affected side was the dominant side. * based on Multiple Imputation. ** based on complete-case analysis.

## Data Availability

For detailed data the corresponding author can be contacted.

## Supplementary Materials

The following supporting information can be downloaded at: https://www.mdpi.com/article/10.3390/jcm13051381/s1, Table S1: Summary of adverse events and following treatment.
