# Supplementary material for: All-Suture Anchor vs. Knotless Suture Anchor for the Treatment of Anterior Shoulder Instability—A Prospective Cohort Study
Source: J Clin Med. 2024 Feb 28;13(5):1381. doi: 10.3390/jcm13051381 (PMC10934154; doi:10.3390/jcm13051381)
Supplement: Supplementary file 1 [file jcm-13-01381-s001.zip › jcm-2868981-supplementary.pdf]

Supplementary Material

Table S1. Summary of adverse events and following treatment

	**SA group**	**KA group**
**Type of event**	Traumatic recurrent dislocation *n=4*	Traumatic recurrent dislocation *n=1*
	Atraumatic recurrent dislocation *n=1*	Atraumatic recurrent dislocation *n=0*
**Treatment**	Revision arthroscopic soft-tissue stabilization *n=3*	Revision arthroscopic soft-tissue stabilization *n=1*
	Revision shoulder stabilization with iliac crest bone augmentation *n=1*	
	Conservative *n=1*	
